# Tool Geometries and Design of Friction Stir Spot Welding (FSSW) Tools and Effect on Weld Properties—A Comprehensive Review

**DOI:** 10.3390/ma18143248

**Published:** 2025-07-10

**Authors:** Aravinthan Arumugam, Animesh Kumar Basak, Alokesh Pramanik, Guy Littlefair

**Affiliations:** 1 School of Mechanical Engineering, Engineering Institute of Technology, Perth, WA 6005, Australia; aravin.arumugam@eit.edu.au; 2 Adelaide Microscopy, Adelaide University, Adelaide, SA 5000, Australia; animesh.basak@adelaide.edu.au; 3 School of Civil and Mechanical Engineering, Curtin University, Perth, WA 6102, Australia; 4 Education and Student Experience, University of Western Australia, Perth, WA 6009, Australia; guy.littlefair@uwa.edu.au

**Keywords:** multi-material design, friction stir spot welding, plastic flow, tool geometry, pin-less tool

## Abstract

The incorporation of multi-material design (MMD) to achieve lightweight vehicles requires Friction Stir Spot Welding (FSSW) to join steel with aluminum, magnesium, or composites. This study investigates the mechanisms, challenges, and performance of FSSW in MMD based on the information available in the literature. It also explores the effect of FSSW tool geometries and design on the spot weld formation and mechanical strength. Larger shoulder and pin diameters increase heat generation during welding. A concave shoulder profile produces a stronger weld compared to flat and convex profiles due to its ability to trap materials and transfer materials to the sheet interface efficiently for the development of a sound weld. Grooves such as Fibonacci and involute, and threads on P-FSSW and R-FSSW tools, also contribute to effective material flow during welding, hence assisting in heat generation. This review also provides recommendations on tool design for FSSW, P-FSSW, and R-FSSW.

## 1. Introduction

**Figure 1:**
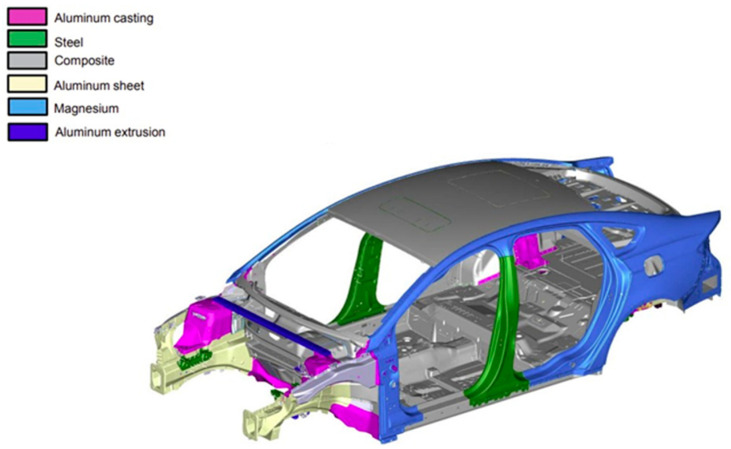
Multi-material design (MMD) of automotive Body-in-White (BiW).

Automotive Body-in-White (BiW) lightweight design has been a priority of automotive manufacturers in recent years. Lightweight design offers advantages such as improved fuel consumption for internal combustion engine vehicles (ICEVs), automotive structural crashworthiness, increased automotive performance for electric vehicles (EVs), hybrids, and ICEVs, minimized impact on the economy and environment, and reduced resources for manufacturing [1,2,3,4]. A 10% mass reduction in automotive vehicles due to lightweight design reduced fuel consumption by 5–7% [5]. For EVs, a 10% reduction in weight improves EVs’ driving range by approximately 14% [6]. The use of a lightweight design in the manufacturing of about 500,000 automotive wheels per year can reduce carbon emissions from manufacturing by 2.56 × 10^7^ kg [3]. A 25% reduction in automotive weight will potentially conserve about 250 million barrels of crude oil per year [7]. Multi-material design (MMD) of automotive BiW is a common strategy that automotive manufacturers consider for achieving lightweight design in the automotive industry. MMD involves the optimized selection of different materials, apart from the conventional steel used for various parts of the automotive BiW, as shown in Figure 1.

The main materials considered in MMD are high-strength steels (HSSs) and advanced high-strength steels (AHSSs), aluminum, magnesium, polymer, and composites. Resistance spot welding (RSW) has been the traditional joining technique in the steel-based BiW, with a BiW comprising on average of around 2000–5000 spot welds [8]. RSW was commonly used in the automotive joining process due to its (i) low cost, as no filler metal is required, (ii) fast operation, and (iii) ease of automation when fitted on a robotic manipulator. RSW involves the localized melting and solidification of a volume of material at the sheets’ interface due to the Joule heating generated by resistance to current flow through the metal sheets. The process uses two water-cooled copper electrodes that generate clamping force on the sheets to be joined. The incorporation of MMD to achieve lightweight vehicles has imposed a challenge in using RSW to join steel with aluminum, magnesium, or composites. The vast difference in thermal conductivity between steel and aluminum required higher energy consumption to join these metals together using RSW, and the formation of a brittle intermetallic compound (IMC) during RSW led to a weak spot weld joint [9]. RSW was also found to be unable to join steel with carbon fibre-reinforced polymer (CFRP) [10], as the former is an insulator [11].

**Figure 2:**
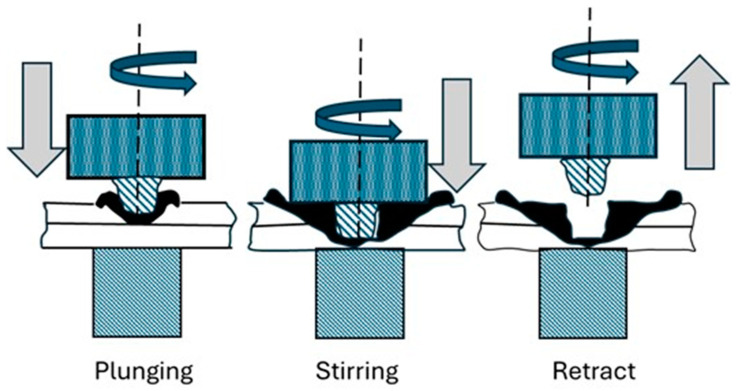
Stages of Friction Stir Spot Welding (FSSW) process.

The Welding Institute (TWI) introduced Friction Stir Welding (FSW), a solid-state welding method to accommodate the need to join different materials in MMD. A variant of FSW, Friction Stir Spot Welding (FSSW), is considered a substitute for RSW for forming challenging joints between soft metals such as aluminum and magnesium with polymers and composites. Unlike RSW, where heating is produced due to the material’s resistance to current flow, in FSSW, heat is produced due to friction created between the material and a rotating tool. FSSW was originally invented by Mazda Motor Corporation in 1993 and has wider application in the aviation and automotive industries [12]. The FSSW process comprises three stages: plunging, stirring/dwelling, and retracting, as shown in Figure 2.

**Figure 3:**
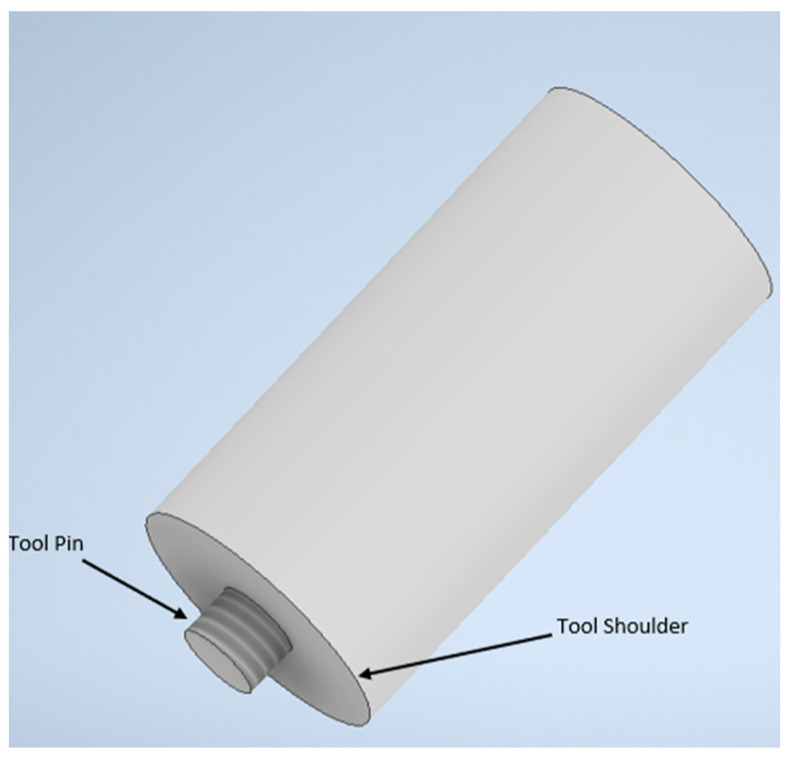
Shoulder and pin of a FSSW tool.

The plunging phase involves the rotating tool being forced into the sheets of material to be joined until the tool’s shoulder encounters the top material sheet. The stirring phase involves the rotating tool reaching the predetermined depth in the materials to be joined. Frictional heat is generated, and the material closer to the tool is heated, softened, and forms a solid-state spot weld at the sheets’ interface. The third phase involves the tool retracting from the joined materials. A tool used in the conventional FSSW comprises two components: the tool shoulder and pin or probe, as in Figure 3.

A keyhole is observed in the joined materials due to the tool design, which significantly reduces the strength of the joint [13]. Conventional FSSW, however, leads to different variants, mainly determined by the changes in the tool design and geometry. Refill FSSW was developed by the Helmholt-Zentrum Geesthacht/Institute of Materials Research—Germany in 2004 to eliminate the keyhole developed in conventional FSSW. Refill-FSSW (R-FSSW) uses a tool that is made from three different components: probe/pin, shoulder/sleeve, and clamping ring. The relative motion between the probe and the shoulder refill the keyhole with material before the tool is retracted from the sheets that are joined. R-FSSW is able to eliminate the keyhole and improve the spot weld strength [14]. This somewhat resembles the friction stir processing (FSP) of materials [15,16,17]. However, the process was found to be more complicated in terms of tool design compared to traditional FSSW [18].

**Figure 4:**
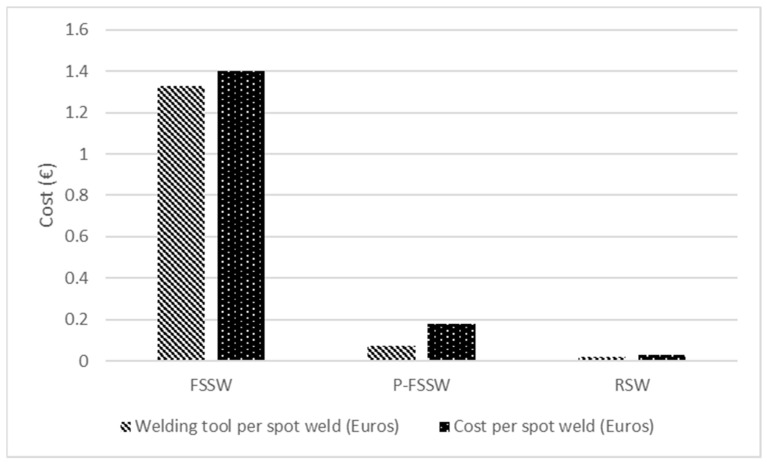
The cost of one spot weld for FSSW variants and RSW, and the cost breakdown for each process. Adapted and redrawn based on the information in the literature [20].

Pin-less FSSW (P-FSSW) uses a tool with a shoulder with features on the face of the shoulder, but without a pin. P-FSSW was reported to reduce the keyhole and hook defect associated with conventional FSSW and, in turn, lead to improved weld strength with a simpler tool design, compared to conventional FSSW [19]. Figure 4 compares the tool costs between FSSW, P-FSSW, and RSW. Figure 4 shows that the welding tool covers 95% of the overall cost of FSSW, 40% of the overall cost of P-FSSW, and 60% of the overall cost of RSW. 

The cost analysis was based on the joining of similar and dissimilar joints of DP590 sheets with thickness 1.75 mm and DP780 sheets with thickness 1.5 mm. The tools used in the study [20] were as follows: (i) tungsten–rhenium alloy FSSW tool of 15.5 mm shoulder diameter with concave profile, and truncated cone pin of 4 mm diameter with pin lengths of 1.75 mm and 1.5 mm; (ii) tungsten–rhenium alloy P-FSSW tool of 15.5 mm diameter with flat profile; and (iii) Cu-Cr-Zr electrodes with 6 mm tip diameter. However, Figure 4 also shows that the cost of RSW per spot weld is the lowest compared to the per spot weld cost of FSSW and P-FSSW. In another study, the tooling cost for R-FSSW per spot was found to be two orders of magnitude greater than the tooling cost of RSW per spot, but that R-FSSW is more energy efficient than RSW, with a difference of 195 kWh per 5000 spot welds [21]. The higher tool cost of FSSW is related to the tool design and geometries, and the high tool wear. Common materials used for the FSSW tool are 30 HRC hardened steel, AISI H13 tool steel, M2 high-speed steel, stainless steel [22], tungsten carbide, 34CrNiMo6 steel, and WC-Co alloy/coated tools [23,24]. The coefficient of friction and thermal conductivity of different tool materials influence the heat generation during welding, hence affecting the weld strength [25]. The front section of the shoulder of the R-FSSW tool, which comes in contact with the materials to be joined and the clamping ring, was subjected to significant wear compared to the probe [26,27]. The shoulder wear reduced the joint area, leading to lower joint strength and increased the hook height [27].

## 2. Scope of This Review Paper

The tools used in FSSW and its variants are more complex than for RSW. The tool shoulder is responsible for the generation of heat during welding due to friction between the tool and the workpiece, while the tool pin is responsible for heat generated due to plastic deformation within the materials, breaking of the oxide layer, and material flow during the stirring action [28,29,30]. The shoulder contributes to the heat generation more than the pin, as the diameter of the shoulder is generally two or three times greater than the diameter of the pin [31]. The FSSW tools have different attributes in terms of shoulder face shapes, shoulder face features, pin shapes, and pin features, as given in Table 1.

Similar to the electrode in RSW, FSSW tools contribute to the spot weld strength and weld defect [32]. Even though authors have reported the effect of FSSW tool design on weld formation and weld strength, limited work has been observed to critically compare the effect of different tool shapes and features on weld growth and mechanical strength. The scope of this review paper investigates the impact of each shoulder and pin shape and feature on spot weld formation and weld strength. It will scientifically and systematically analyse the shoulder and pin geometries to provide a comprehensive analysis of the effect of the design and geometries of the FSSW tool on material flow, weld formation, and weld strength. The review intends to provide shape- and feature-relevant tool design recommendations that can be used by researchers and manufacturers to successfully produce quality welds for different combinations of materials.

## 3. Welding Strategies and Parameters

The FSSW process involves process parameters such as tool rotational speed, dwell time, plunging depth or rate, and tool geometries/profiles. Most work has reported tool rotational speed to have the highest effect on spot weld strength and hardness [33,34,35,36]. However, other studies have also reported pin profiles [37,38,39,40] or plunge depth/rate [38,41,42,43] to make a high contribution to weld mechanical properties, followed by tool rotational speed. These studies show that tool design is directly related to the tool rotation and the plunging depth. Increasing the tool rotation speed increases the friction between the tool and the surrounding materials. This increase in friction generates the required temperature rise to create the plastic deformation of materials and the formation of the weld joint. Higher tool speed will also lead to a lower weld strength due to excessive tool penetration, leading to reduced top sheet thickness [44]. A plunge depth or rate increase will increase the volume of plastically deformed material, hence expanding the stir zone or joint area. The pin profile and shoulder diameter affect the plunge depth, leading to the variation in the amount of material being moved and contributing to frictional heating during welding [45,46].

The tool’s rotational motion (rotational speed) and translational motion (plunging rate) within the materials to be joined are responsible for the heat generated during this process [39]. The heat generation of a tool is a function of the shoulder diameter, pin diameter, and pin length [33,47,48], as in Equation (1):

(1) $$Q_{\mathrm{total}}=K\;\times \;\omega \;\times \;t(R_{shoulder}^{3}+3R_{pin}^{3}H_{pin})$$

where Q—the heat input during FSSW; K—friction-related heat generation coefficient that depends on material properties such as friction coefficient and thermal conductivity; ω—rotational speed of tool; t—dwell time; R_shoulder_—tool shoulder radius; R_pin_—tool pin radius; and H_pin_—pin height.

**Figure 5:**
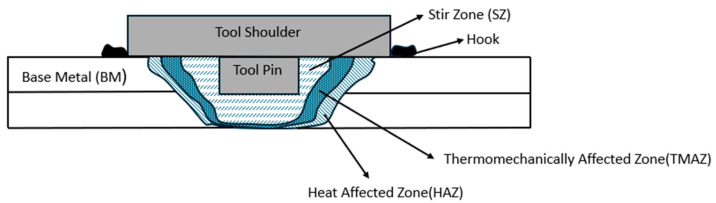
Microstructural zones of a FSSW weld joint.

The heat generated during the FFSW produced the weld joint. The weld joint of a FSSW is comprised of four distinct microstructural zones: the stir zone (SZ), the thermo-mechanically affected zone (TMAZ), the heat-affected zone (HAZ), and the base metal (BM), as in Figure 5. The SZ is the area under the shoulder and periphery of the pin that is subjected to extreme plastic deformation and frictional heat during welding, causing dynamic recrystallisation and the formation of refined equiaxed grains. TMAZ is an area closer to the SZ, which is more subject to the influence of the deformation due to tool rotation rather than heat, leading to the formation of elongated grains. The HAZ is the area after TMAZ, which has the coarsest grains of all three areas, as it is only subjected to welding heat during welding, with no significant plastic deformation. The tool diameter ratio (D/d), where D is the shoulder diameter and d is the pin diameter, influences the size of grains formed at the SZ, TMAZ, and HZ [49]. The largest welded joint or the SZ area and the highest hardness of the SZ are related to higher joint strength, and FSSW joint failure occurs at the TMAZ/HAZ due to the coarser grains [50,51,52].

## 4. Effect of the Tool Shoulder

During the plunging phase, the tools’ vertical force and torque increased with their shoulder diameters. When the shoulder comes into contact with the top sheet, a larger axial pressure is generated, eventually dropping due to the softening of materials under the shoulder. A higher temperature develops under the shoulder compared to the surrounding of the pin during welding [53].

**Figure 6:**
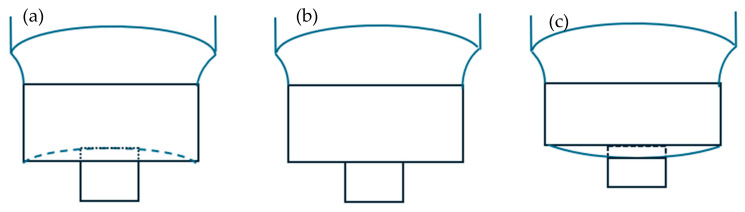
Tool shoulder profiles (**a**) concave, (**b**) flat and (**c**) convex.

Generally, the larger the shoulder diameter, the larger the joint strength [54,55], with the exception being if overheating occurs. The tool shoulder is mainly made of three different profiles: flat, concave, and convex, as shown in Figure 6.

The force at the initial plunging stage is higher for the convex shoulder than the concave shoulder for the same welding condition [56]. The convex shoulder tends to move material outwards away from the pin, requiring higher force and torque to cause material deformation and flow. The convex shoulder also comes into contact with the material earlier, hence heating the material earlier compared to the concave and flat shoulders. The effect of the convex shoulder with different radii showed that a radius of 22.5 mm reduced the dwell time to produce acceptable welds by three times compared to a radius of 15 mm [57]. The concave shoulder traps materials during welding, leading to higher weld mechanical properties compared to flat and convex shoulder profiles [56,58,59,60]. The greater effect of top sheet thickness in the concave shoulder compared to flat and convex shoulders provides the greatest resistance to crack propagation through the sheet thickness; hence, the concave shoulder produced the strongest weld, and the convex shoulder produced the weakest weld [61]. Aluminium alloy and carbon fibre-reinforced polyamide were joined using four different tools: 15 mm diameter flat shoulder, 20 mm diameter flat shoulder, 20 mm diameter concave shoulder, and 20 mm diameter multi-ring shoulder. The concave shoulder tool decreased the thermal decomposition defect compared to flat shoulder tools and had a larger effective joining area and uniform interface temperature compared to the multi-ring shoulder tool [62]. The concave shoulder tool was found to have the highest joint strength because the tool produced a more uniform temperature distribution, the largest covalent bonding area, and improved mechanical interlocking compared to the other tools.

The weld strength improved with an increase in concavity angle, as an increase in angle led to more materials being trapped under the shoulder [59], and this later participated in heat generation via friction with the recommended minimum of 4.5° shoulder concavity angle [63,64]. The comparison between concave and convex shoulders with the cylindrical pin of the same pin diameter and length showed that the downward plastic material flow is more prominent in the concave shoulder compared to the convex shoulder [65]. Therefore, better material mixing occurs between the downward flow of material due to shoulder pressure and the upward flow of material due to pin rotation. Due to this mixing of material, hook formation was observed in the concave shoulder, and no hook was observed in the convex shoulder. In joining aluminium alloy and CFRP, three different tools were studied: flat shoulder tool with 2 mm diameter pin; a tool with a 3 mm deep circular hollow on the shoulder (90° angle) without a pin; and a tool with a 3 mm deep circular hollow on the shoulder (90° angle) with a pin 2 mm diameter pin. The tool shoulder diameter for all three tools was maintained at 10 mm. Even though the tool with the circular hollow without a pin produced the highest weld heat input, the tool with the circular hollow with a pin was able to suppress the generated heat and spread the heat effectively to prevent overheating at the joint area [66]. Hence, the tool with a circular hollow with 90° angle and a pin was able to produce the largest weld area, and it welded with the highest strength compared to the other tools. The produced weld area and weld strength improvements were 12% and 4% respectively, compared to the flat shoulder with the same pin size.

**Figure 7:**
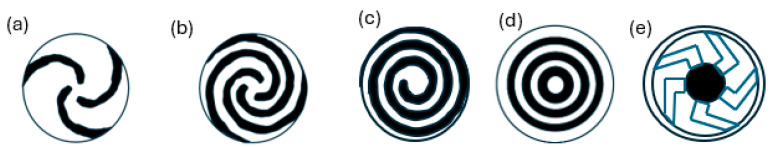
Tool grooves geometries: (**a**) Archimedes groove, (**b**) involute groove, (**c**) scroll groove, (**d**) annular groove, and (**e**) 5-L groove shapes. Adapted and redrawn based on information in the literature [69,70,71,72].

In P-FSSW, the hook did not form as compared to a concave shoulder with a tapered pin due to the lateral flow of the material during welding [19]. However, it was also reported that the upward flow of material is limited in this tool, leading to a poor strength weld compared to a concave pinned tool. A study with similar Al-Al, similar Cu-Cu, and dissimilar Al-Cu using a pin-less tool and flat shoulders with pins with the same shoulder diameter showed that the pin-less tool produced welds with greater strength compared to the tools with pins. The pin-less tool in this work was made from embedded Cu and Al rods in the centre. Due to higher friction at the centre of the tool, greater heat was generated, leading to bigger welds and stronger joints. Lower thickness reduction of the top sheet, a uniform strain below the tool, highly concentrated heating, and abnormal hook geometry that delayed crack propagation resulted in a higher weld strength in the pin-less tool [67]. The effect of a pin-less tool with two types of grooves on flat shoulders and a concave tool on spot weld growth and strength shows that the downward force increased with plunge depth, and the forces were greater on grooved flat shoulders compared to concave shoulders [68]. Of the grooves used, the Archimedes groove shoulder (Figure 7a) showed more effective material flow compared to the involute groove shoulder (Figure 7b) and concave shoulder. The use of grooves generally promotes better-plasticised material flow through the grooves, downwards to the sheet’s interface, and different types of grooves facilitate the flow differently. The Archimedes groove created the largest SZ; however, it was prone to hook formation with the largest height at the interface. The concave tool, even though it demonstrated moderate material flow and hence a moderate SZ size, had better forging force control, affecting hook formation and leading to a smaller hook height for the same welding condition as the Archimedes groove flat shoulder.

The basin-shaped weld profiles reported by Chu et al. [68] were also reported by Guishen et al. [69], who studied P-FSSW with a flat tool shoulder and three different types of grooves: Archimedes, involute, and scroll (Figure 7c) on the flat shoulders. The grooves on the shoulders enhanced the stirring effect, leading to this profile on spot weld joints. However, this study contradicts the results of Chu et al. [68] between Archimedes and involute grooves by reporting that the involute grooved shoulder tool is the optimum tool to be used according to the results from the Taguchi parameter optimisation analysis. This study also reported that a groove-less flat shoulder has the least efficiency in material flow during welding due to decreased stirring effect. Another study that investigated P-FSSW with tools that have flat shoulders but different features—no groove, annular groove (Figure 7d) and involute groove—also reported the basin-shaped weld profile for the involute groove as the earlier to work [70]. The tool with an involute groove was again found to be the tool that produced the most favourable weld formation. The tool with an involute groove produced the most severe plastic deformation during welding, causing the sheets to be bonded via mechanical interlocking. The Fibonacci spiral curve groove, involute spiral groove, and Archimedean spiral curve groove P-FSSW tools that were used to join copper sheets revealed that the Fibonacci groove produced the most efficient material flow within the weld region compared to the involute and Archimedean grooves [71]. As reported by [69], this work also reported that the material flow using involute grooves is better than that of Archimedean grooves. The reason for this might be the difference in plunge depth. In [68], the plunge depth was 0.5 mm, while in [69,71], the plunge depth was 0.3 mm; hence, tool plunge depth affected the weld development of different grooves [69]. The SZ width and depth of Fibonacci grooves were the greatest of all three tools, followed closely by the SZ width and depth of the involute’s grooves. Hence, due to the advantage of Fibonacci grooves in efficient material flow guidance, the welds developed using this tool showed the highest weld strength, followed closely by the welds developed using involute grooves. Scroll grooves on a tool with a shoulder concavity of 10° and another tool with 5 L-shaped grooves (Figure 7e) with 0.5 mm depth on the shoulder surface were used to join aluminium alloy sheets. Observation of the microstructures formed using both tools showed that the scroll grooves produced a uniform material stirring compared to the 5 L-shaped grooves, as the latter’s sharp corners caused severe stirring at the SZ [72]. This severe stirring produced greater plastic deformation, leading to the formation of a wider and deeper SZ, and the 5 L-shaped grooves tool produced stronger welds compared to the tool with scroll grooves.

**Figure 8:**
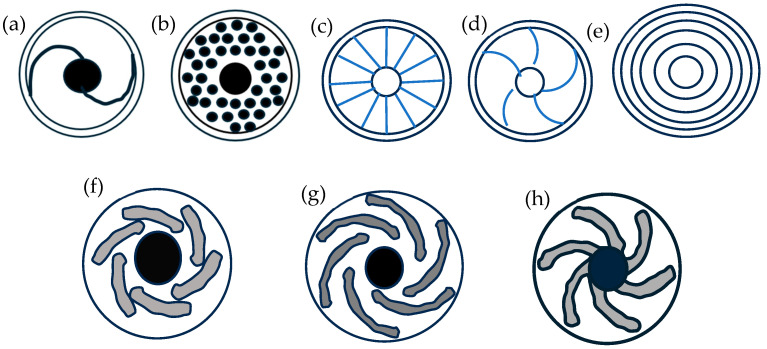
Tool geometries: (**a**) scroll, (**b**) ridged, (**c**) knurled, (**d**) grooved, (**e**) concentric circles, (**f**) short flute wiper, (**g**) long flute wiper, and (**h**) proud wiper. Adapted and redrawn based on the information in literature [74,75].

A comparison of different shoulder geometries was featured in Figure 8 [73]. The P-FSSW tool with concentric circles produced the most heat and plastic deformation during welding, leading to more recrystallisation in the microstructure. Concentric circles and scrolled shoulder features also produced smaller grains compared to the other features. However, the ridged profile produced the weld with the highest mechanical joint.

A featureless tool, short flute wiper tool (Figure 8f), long flute wiper tool (Figure 8g), fluted scroll tool, and proud wiper tool (Figure 8h) were also studied [74]. The flute wiper tool was found to be the tool that generated the highest weld temperature, while the proud wiper tool produced the lowest weld temperatures. Therefore, the flute wiper tool produced the highest weld strength, and the proud wiper tool produced the lowest weld strength. The work reported that material flow between the top and bottom sheets depends on weld time and tool surface features. The flute and scroll features were successful in trapping and driving the material flow toward the centre of the weld. In a study joining Al with CF/PA6 using a P-FSSW flat tool and a tool with a recess, the recessed tool suppressed the downward plastic deformation flow of Al into CF/PA6, in turn suppressing the porosity density defects at the CF/PA6 side. The recessed tool also concentrated the heat generation in the middle of the joint and generated less heat [75]. Hence, the recessed tool produced joints with higher strength compared to the flat tool.

**Figure 9:**
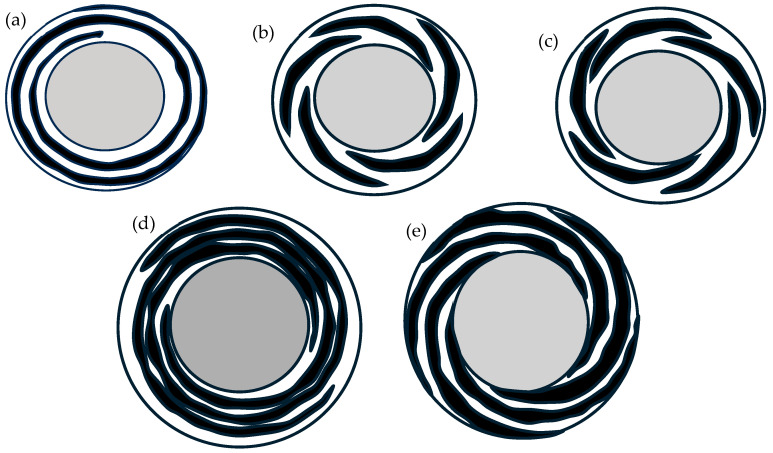
R-FSSW shoulder features: (**a**) continuous spiral, (**b**) 5-incised spiral section with same direction rotation as the shoulder (tool) rotation, (**c**) 5-incised spiral section with opposite direction rotation as the shoulder (tool) rotation, (**d**) two spirals rotated 180° to each other, and (**e**) 5 pieces of spiral symmetrically arranged around the tool rotation axis. Adapted and redrawn based on information in the literature [78,79].

In a study of R-FSSW [76,77], comparisons between standard flat shoulders and modified shoulders (45° chamfered shoulder and three trapezoidal grooved shoulders) showed that the modified shoulders improved the flow of material, increased heat generation, improved the effect of hooks by reducing the stress concentration effect, and eliminated defects at the SZ/TMAZ during the refill stage. At the SZ/TMAZ interface and the SZ, highly refined grains were produced with modified shoulders compared to the standard shoulder, meaning that modified shoulders direct more of the pin’s refill force to the weld periphery. The improved mixing of material and higher deformation energy in the modified shoulders also increased the tensile strength of the weld compared to the welds produced using the standard shoulder. Figure 9 shows different shoulder front face features for R-FSSW: continuous spiral; the 5-incised spiral section with the direction of the 5 spiral sections in the same direction as the shoulder (tool) rotation; and another tool, with the direction of the 5 spiral sections in the opposite direction to the shoulder (tool) rotation.

In comparison with the shoulder without any feature, the shoulders with a continuous spiral and the 5 spiral sections in the same direction as the shoulder rotation showed a better mixing of materials during welding [78]. The 5 spiral sections in the opposite direction of tool rotation showed noticeable material flow disturbance, which negatively affected the weld strength. Łogin et al. [79] also studied three different shoulder front face features for R-FSSW: continuous full-grooved spiral, two spirals rotated 180° to each other, and 5 pieces of spirals symmetrically arranged around the tool rotation axis. The study reported that the continuous grooved spiral demonstrated better mixing of materials at the sheet’s interface compared to the other two features and had the highest tensile shear failure load.

The results obtained for R-FSSW with standard sleeves and sleeves with threads and grooves showed that threads and grooves improved the mixing of material during welding [80]. The difference in material flow in the sleeves with features compared to the standard sleeves reduced the height of the hook, with the lowest hook obtained being in the shoulder with grooves. The height of the hook also governed the tensile shear strength of joints made with these shoulders, with the lower height of the hook in the grooved sleeve producing a higher tensile strength of welds. A sleeve with a smooth surface does not accelerate the material surrounding it during the refill stage, hence reducing the maximum velocity of material flow [81]. Grooves on sleeves accelerate the flow of material in the vertical direction, which potentially forms hooks. The material flow surrounding the sleeve can also be accelerated using a larger-diameter sleeve; however, it will potentially increase the peak temperature and affect the heat-affected zone. The study also indicates that a sleeve with scrolled grooves at the bottom is better than a sleeve with the bottom with concentric circles in terms of increasing bonding area. Shoulders that have tapered shapes at the end face with three different locations—inner shoulder side, centre of shoulder; and outer shoulder side– showed a different ability to remove zinc coating when joining aluminium alloy and galvanised steel [82]. The tool with a shoulder with a 0.2 mm taper at the inner side showed the highest ability to remove galvanisation and improve the joinability between aluminium alloy and galvanised steel. This led to the development of welds with higher tensile strength. The shoulder with the taper at the centre failed to remove the galvanised layer at the interface of both metals, leading to welds with lower strengths.

## 5. Effect of Tool Pin on Weld Joint Strength

**Figure 10:**
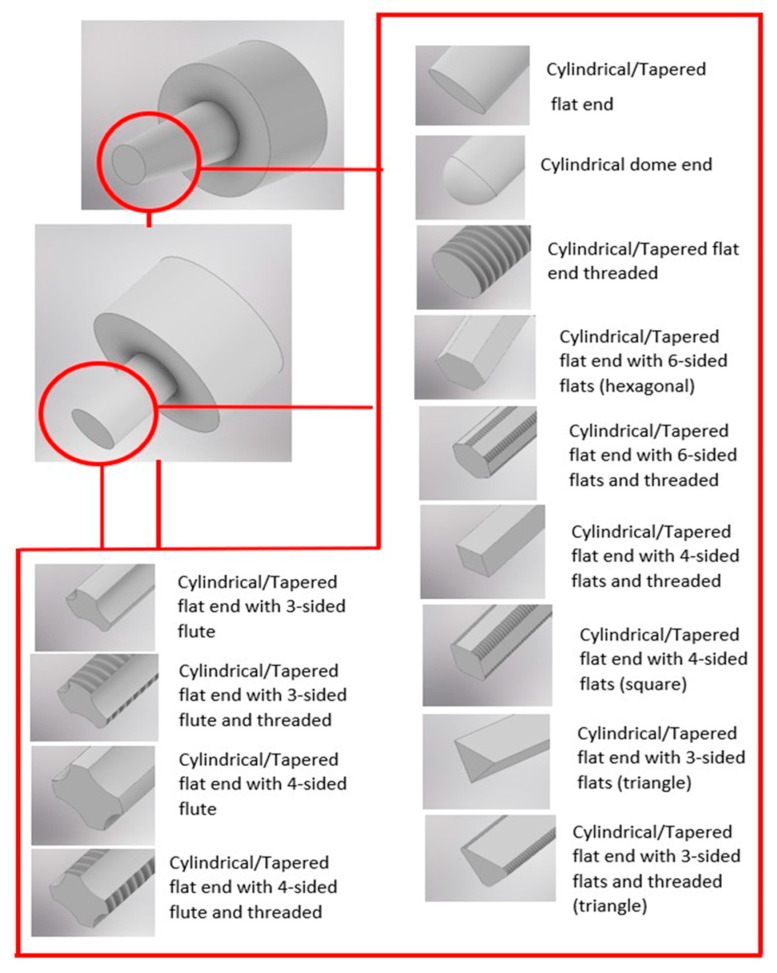
FFSW pin/probe shapes and features.

An optimisation study using the Taguchi method confirmed that the pin profile has a significant effect on the tensile shear strength and bending strength of aluminium alloy spot weld joints, with 65.6% and 207% contribution, respectively, compared to plunge depth, spindle speed, and dwell time [38]. Figure 10 shows all the pins/probes used in the FFSW process. An increase in pin diameter creates a larger grain size in the HAZ [83]. The larger diameter causes more material mixing, leading to more frictional heat generation, which is transferred to the HAZ to form coarse grains. In terms of microhardness, an increase in pin diameter decreases the microhardness of HAZ but increases the microhardness of SZ. During plunging, the axial force on the tool depends on the base area of the tool; the hexagonal pin with the highest base area has a higher axial force compared to the triangular pin, which has the lowest base area. The forces for taper cylindrical and square were intermediate due to similar base areas [31]. The triangular pyramid pin was reported to be the optimised pin for the tensile shear strength of the spot weld joint, and the square pyramid pin for the bending strength of the spot weld joint. A study on four different pin shapes, cylindrical, triangular, tri-flute, and flat pin for ABS plates [84], with the same shoulder dimensions showed that the triangular pin produced the largest bond diameter, as the triangular pin can displace greater material under the shoulder, leading to more material softening due to the frictional heating. The study also identified that the material displaced by the cylindrical pin was similar to the triangular pin, but that the cylindrical pin cannot flow the material under the shoulder as effectively as the triangular pin. The highest weld strength, due to the highest bond area, was exhibited by the flat pin due to the smallest keyhole size, and the weakest weld strength was shown by the cylindrical pin due to the biggest keyhole size and smallest bond area.

The use of cylindrical and conical pins for aluminum alloy also revealed a smaller-sized keyhole in the middle of the specimen, and a higher bond area (wider SZ) formed with the conical tool compared to the cylindrical tool [85]. The conical pin tool produced higher bonding areas compared to the cylindrical tool, leading to higher tensile strength. This study also showed the different effects of both pins on hook formation and crack propagation during the tensile test, with the hook geometry causing the crack to propagate into the SZ in cylindrical pin welds. A conical or, at times known as, tapered pin has a higher welding force compared to cylindrical, cylindrical threaded, and square pins [63]. This high force creates a high friction force around the pin, leading to higher temperatures during welding and the thickest and strongest weld compared to the other pins. The cylindrical pin remains as the pin that produced the weakest weld.

The study joining DP780 steel with triangular and tapered pins showed that the triangular pin produced welds with a strength 50% higher than weld strength produced using a tapered pin for the same welding condition [86]. This difference in strength was due to the characteristics of the hook and the difference in crack propagation. Unlike the tapered pin, where the hook moved upwards to surround the SZ and eventually moved downward to the weld bottom, in the triangular pin, the hook moved upwards near to SZ and stopped at the plateau. Welds formed from a triangular pin were found to fail due to tensile failure, while welds from a conical pin failed due to shear strength, with finer grains formed in the SZ by the triangular pin [87]. The triangular pin produces more enhanced material flow pattern and mixing compared to the cylindrical pin, tapered pin, and inversed tapered pin [65]. For a cylindrical pin, larger material mixing occurs at the pin periphery, and for a tapered pin, the material flow occurs along the surface of the tapered pin. The hook formation in the tapered pin is smaller than the cylindrical pin. The triangular pin has a suppressed hook geometry compared to tapered and cylindrical pins because of the strongest material mixing. A comparison between the cylindrical pin and the stepped pin [88] showed that the stepped pin produced higher heat input than the cylindrical pin for the same tool rotation speed, as the stepped pin increased the contribution of the shoulder to total heat. The fully bonded region and hook height are lower in a stepped pin compared to a cylindrical pin due to differences in material flow during welding. Unlike in the cylindrical pin, where the material is extruded from the lower sheet to the top sheet during downward pin movement, the extrusion of material is obstructed in the stepped pin, leading to a shallow penetration into the upper sheet [89]. The cylindrical pin produced joints with higher shear load due to greater fully bonded regions and a finer microstructure compared to the stepped pin. An experiment with seven different stepped tools, including cylindrical and tapered pins, showed that stepped pins with different diameters and heights affect the hook direction and SZ geometry [54]. This work pointed out that weld strengths are also dependent on the tool plunge depth and moderate total weld time. For stepped tools, the shoulder diameter to pin diameter ratio is crucial, and a diameter ratio of between 2.5 and 3 was found to produce welds with superior weld strengths [90].

A comparison between triangular, circular, and square pins used to join aluminium alloys showed that the square pin produced the highest temperature during welding at the TMAZ. The four edges of the square pin, compared to three edges and no edges in the triangular and circular pins, caused higher friction between tool faces and materials, leading to higher heat generation [91]. The work, however, reported that the triangular pin produced the strongest welds compared to the other pins because the mixing of materials during welding was more uniform, leading to better interatomic bonding between aluminium sheets. The square pin developed the lowest weld strength due to non-uniform material mixing during the stirring phase, compared to the triangular and circular pins. A greater intermixing and higher swept volume in triangular and square pins compared to cylindrical pins was also reported by Ibrahim et al. and Garg et al. [14,92]. A work that concentrated on the effect of pin geometry on hook formation pointed out that a hook has three geometrical features: the effective top sheet thickness, effective weld width, and hook height [93]. The cylindrical pin had the greatest effective weld width and hook height. The conical pin had the highest effective top sheet thickness. The hexagonal pin had the lowest of all three geometries. The effective weld width determined the weld strength. The higher effective weld width resulted in the higher weld strength. The weld strength was higher with the cylindrical pin, whereas the hexagonal pin led to the lowest weld strength. A weld strength comparison between a cylindrical pin and a tapered/conical pin or triangular pin found results opposite to those observed in [63,84,86,91]. The process parameters seem to play a role in this case. The dwell time played a role in increasing the effective weld width for the cylindrical pin, producing a higher weld strength than the triangular pin. Similarly, at a lower plunge depth of below 2 mm, the cylindrical pin showed higher maximum failure load compared to the triangular pin for both an aluminium–bulk metallic glass dissimilar joint and magnesium–bulk metallic glass dissimilar joint. After a plunge depth of 1.9 mm, the failure load for the triangular pin increased, and the failure load became similar to the failure load achieved using a cylindrical pin at 2 mm plunge depth. At a lower plunge depth, the triangular pin had a dominant cutting effect, and at higher plunge depth, the friction stirring effect had a higher effect. The plunge depth did not affect the cylindrical pin [94].

The use of a 4 mm diameter cylindrical pin and a 2 mm tip diameter tapered pin with a conical shoulder to join polypropylene sheets showed that the cylindrical pin, due to a larger diameter, produced heat at a faster rate at two different rotational speeds compared to the tapered pin. The material flow towards the shoulder was more for the tapered pin, and was eventually pressed downward towards the lower sheet by the shoulder concavity. At the higher speed, the flow of material below the tool shoulder produced a cavity on the top sheet with the tapered tool. Such a problem was not observed with the cylindrical pin due to its lower material flow and flatter material flow under the tool shoulder. The cylindrical pin produced the highest weld strength at a higher speed because the tapered pin produced welds with a cavity, which led to a drop in weld strength. At the lower speed, the weld strength of the tapered pin was higher than that of the cylindrical pin [95]. A similar result was also reported in a study that compared tools with a cylindrical pin, tapered cylindrical pin, and threaded cylindrical pin with thread, where at higher speed, the cylindrical pin produced a higher-strength weld, and at the lowest speed, the threaded cylindrical pin produced the strongest weld [96]. Meanwhile, at the highest plunge time, the cylindrical pin produced a higher strength weld, and at the lowest plunge time, the tapered cylindrical pin produced the strongest weld. A cylindrical pin, a threaded tapered pin, and a cylindrical pin with a 0.5 mm projection showed that the cylindrical pin with a projection created minimal deformation because of tool penetration, as the tool shoulder did not touch the top sheet, unlike the other two pins [97]. The minimal reduction in the top sheet thickness due to an increase in tool penetration depth using a pin with projection caused this pin to create a weld with a wider bond area and higher tensile shear strength.

Materials flow differently for step spiral and off-centre hemispherical pins with concave shoulders when joining aluminum alloy. This also depends on the tool rotation speed. For the step spiral pin, at a lower rotational speed, a larger bond area with a flat hook was produced, while at a higher rotational speed, due to higher heat generation, better material flow caused the distinct hook formation and reduction in bond area. For the three off-centre hemispherical pin features, the asymmetrical arrangement of pins and the hemispherical feature produced lower heat compared to the step pin, leading to an unbonded region at a lower speed and flow of material on the horizontal plane instead of vertical flow at higher speeds [98].

The threaded feature on pins such as cylindrical and triangular pins with M4 left-handed threads affects the performance [99]. For threaded pins, the locus of circulating flow observed in the SZ was composed of a multilayered structure and was more obvious than that of pins without threads. The screw threads transferred more materials from the upper sheet to the lower sheet along the screw threads. However, this was more prominent in the threaded cylindrical pin than in the threaded triangular pin, as the screw threads were discontinuous and incomplete in the triangular pin and this interrupted the material flow in the threaded triangular pin. A similar work using a threaded cylindrical pin and an unthreaded triangular pin reported that the effect of pin geometry was negligible at high rotational speed, as both pins gave similar SZ geometries at high rotational speed [100]. This work also reported that at lower speed and the same plunge depth, the threaded cylindrical pin produced a greater SZ compared to the unthreaded triangular pin, as the threaded features increase the flow of material during stirring. A study on full-threaded and half-threaded conical pins used to join aluminium alloy sheets showed that the peak temperature during welding was higher in the full-threaded pin. The differences in friction area (higher friction area in the fully threaded pin) and material flow were the reason for the difference in temperature between these tools.

With a full-threaded pin, more material flows plastically towards the lower sheet, leading to a wider SZ at the bottom sheet [101]. Experimentation has shown that the weld bonding width was greater in a half-threaded pin due to a shorter flow path, lesser frictional energy loss, and greater velocity; hence, welds made from a threaded pin had greater cross-tension failure strength compared to a full threaded pin. The effect of threading location on a 5 mm diameter cylindrical pin with M5 right-hand threads showed that threads closer to the shoulder allow upper sheet materials to flow downwards to the interface, leading to better material mixing compared to threads in the middle of the pin length and at the tip of the pin [102]. The different threading locations on pins of the same length changed the SZ morphology, hence showing the ability to control material flow. A comparison between cylindrical threaded pins, three flat with a 0.5 mm threaded pin and three flat with a 0.7 mm threaded pin, showed high failure load in spot welds produced using the three flat 0.7 mm threaded pin due to it producing greater bond width; this tool also modified the hook curvature, causing the hook to bend away from the tool axis [103].

In joining aluminum alloy and low-carbon steel, the use of pin lengths of 2.3 mm, 2 mm, and 1.7 mm with the same shoulder diameters and conical pins showed that the highest temperature during welding was observed with the longest pin of 2.3 mm, which was due to the removal of the galvanized coating on the steel, leading to an increase in friction [104]. The use of a longer pin that penetrates the bottom steel plate therefore breaks the galvanized coating. The zinc particles play an important role in increasing the weld strength by forming an intermediate layer between the plates. The long pin tool in this study produced a nugget pullout failure, while the short and medium pin tools produced interfacial failures. A similar result was also reported in [105], which used a pin-less tool, a tool with a tapered pin with a 5 mm tip diameter and 0.3 mm length, and a tool with a tapered pin with a 9.6 mm tip diameter and 0.4 mm length. The tapered pin with a 9.6 mm diameter and 0.4 mm length produced the optimized weld strength, with a lower distance from the pin tip to the bottom of the lower sheet. This tool produced the thickest IMC at the interface of the sheets due to greater diffusion during welding, leading to greater bonding. The work pointed out that the maximum thickness of the IMC should be 0.8 μm, after which a thicker IMC will lead to weaker joints. In this work, the IMC thickness was 0.5 μm.

A micro FSSW study [106] reported a longer pin length, where the shoulder not coming into contact with the top sheet produced a lower temperature. This work compared a pin-less tool and three other tools with 5 mm shoulder diameters but different pin lengths (250 μm, 450 μm, and 650 μm). The tool with a 450 μm pin length produced the highest temperature and strongest weld, while the 650 μm pin length produced the lowest temperature because in the former tool both the shoulder and pin were involved in heat generation, while in the latter tool only the pin was involved in heat generation. In a similar and dissimilar joining of aluminum and copper using a pin-less tool, a tool with flat shoulder and 3.3 mm diameter cylindrical pin (two lengths of 0.2 mm and 0.4 mm), and a tool with flat shoulder and 4.95 mm diameter cylindrical pin (two lengths of 0.2 mm and 0.4 mm), the use of pinned tools increased the plunge depth, leading to a severe reduction in the thickness of the top sheet, higher strain accumulation surrounding the pin, and the formation of a hook that promoted joint failure through crack propagation. The work reported that an increase in pin diameter (3.3 mm to 4.95 mm) and pin length (0.2 mm and 0.4 mm) increased the SZ due to an increase in material diffusion causing an increase in the bond area. However, the increase in pin length decreased the weld strength, as this led to a reduction in the top sheet thickness [67]. The use of a pin-less tool and tools with 0.4 mm and 0.6 mm pin lengths to join aluminium sheets also reported that the tool with the longer pin improved the fatigue strength of the welds [107]. The increase in pin length increased the weld size, leading to higher tensile shear failure; however, shoulder indentation caused a reduction in the top sheet thickness. The dependence of the cross-tension strength on probe length changes is, however, unclear [108]. The effect of different pin lengths on SZ shapes is discussed in [109]. A pin length of 3.5 mm formed a SZ which was elliptical in shape. However, a pin of 4 mm length, which generated greater heat, also affected the material flow around the pin and below the shoulder, leading to a distorted elliptical SZ. The study also reported that variation in pin length affected the grain size in the SZ, but that the microstructures at the HAZ and TMAZ were not affected.

## 6. Discussion and Recommendation

**Figure 11:**
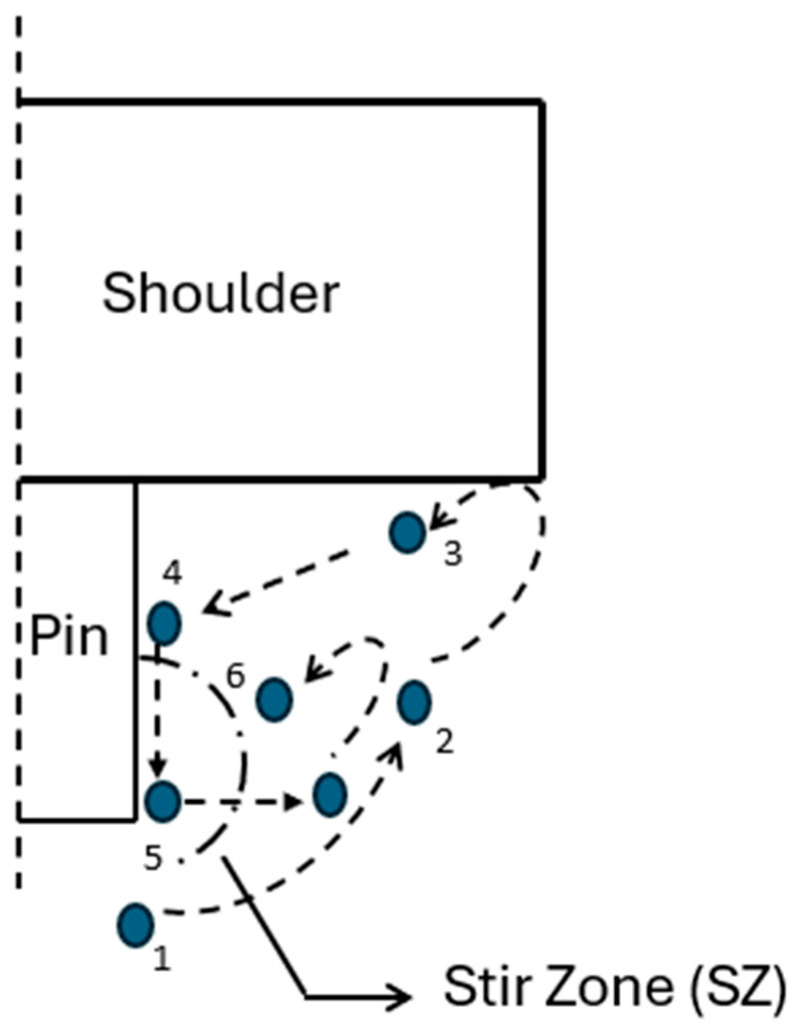
Material flow in the FFSW process. Adapted and redrawn based on the information in the literature [110].

The design of an FSSW tool shoulder and pin directly affects the material flow during the welding process. The material flow during FSSW involves the materials below the pin being displaced upwards while the pin penetrates the sheets to be joined. The mix of the lower sheet material and upper sheet material is then displaced downwards when the tool shoulder encounters the upper sheet. This creates a continuous circulation of material due to the movements of the pin and shoulder. Hence, the material flow can be divided into two components: the lower sheet material moving and mixing with the upper sheet material (Point 1–3), and the flow of the mixed materials along the pin (Point 4 and 5), as in Figure 11. These motions will eventually build the SZ (area comprising Point 6) [110,111].

An increase in shoulder diameter increases the material flow circulation, heat generation due to friction, and development of welds with greater strengths. This review indicates that the concave shoulder tool was able to effectively cause material flow along the pin for joint formation compared to convex and flat shoulders, as the concave shoulder concentrated material under the shoulder, creating a good material mix due to a forging effect. The P-FSSW tool had a weaker upward material flow than a tool with a pin. The pin is responsible for the plastic deformation of materials and moving the plasticized materials upwards at the initial stage of the materials’ flow. The absence of a pin in P-FSSW reduced the ability of materials to move upward to the top metal sheet. The use of features on the shoulders of P-FSSW tools assists in improving the material mix, material flow, and heat generation. Grooves assist in improving material flow by accumulating the materials that are driven by the rotating tool within the walls of the grooves [112]. Under the pressure of the tool, the materials in the grooves are pushed downwards, creating material circulation. An absence of grooves reduces the downwards flow of materials, which eliminates the formation of hooks [113]. The downward flow of materials can be improved with the tool’s rotational speed. The degree to which materials flow with the aid of grooves depends on the groove and profile types. Grooves such as Archimedean, involute, Fibonacci, scrolled, and spiral, and profiles such as ridged for P-FSSW, and shoulder chamfer and sleeve threads for R-FSSW, have been shown to improve weld strength due to better material mixing and more heat generation. Fibonacci grooves produced joints with the highest tensile strength, followed by involute grooves and Archimedean grooves, indicating that Fibonacci grooves provide the most efficient.

An increase in pin diameters also increases the friction due to material plastic deformation and material flow, leading to higher heat generation and the development of welds with greater strength. Different pin shapes and profiles also play a major role in improving material mixing during the FSSW process. Pins with edges, such as hexagonal, pentagonal, square, and triangular pins, were found to have better material-mixing abilities, as materials are plasticized due to shearing and friction compared to circular tools such as cylindrical and tapered pins with no edges. The hexagonal pin was found to produce the strongest welds, with more edges being able to produce more shearing of materials, leading to uniform mixing of materials, higher heat input, and defect-free welds. The triangular pin had a better material-mixing capability compared to the cylindrical and tapered pins. A cylindrical pin has been reported to have a lower ability to displace materials upward to the tool shoulder. The size of the keyhole is crucial in determining the weld strength, and a flat pin has been reported to have the smallest keyhole size and greater bonding area compared to triangular, cylindrical, and tri-flute pins. A similar observation applies between conical/tapered and cylindrical pins, with the conical pin achieving greater weld strength compared to the cylindrical pin due to a smaller keyhole size. The plastic deformation of the materials was greater in the cylindrical pin.

Even though the cylindrical pin has been found to have a less efficient material flow compared to hexagonal and triangular pins, the pin shape was found to be superior compared to the stepped pin. Although the stepped pin produced higher heat input, the pin obstructed material flow. Hence, the cylindrical pin produced a stronger weld compared to the stepped pin. The square pin was also found to produce weaker strength compared to the cylindrical pin, even though the square pin produced the highest temperature during welding compared to the triangular and cylindrical pins due to its four edges. The non-uniform mixing with the square pin resulted in the weld strength of the square pin being lower than that of the cylindrical pin. The cylindrical pin can also be improved in terms of its ability to produce strong welds compared to other pin shapes by incorporating a proper selection of dwell time and plunge depth. Reviews have shown that the cylindrical pin produced stronger welds compared to hexagonal, cylindrical, and tapered pins when used with the correct dwell time and plunge depth.

The use of threaded pins has also been shown to improve material flow and mixing during the FFSW process. The positions of the threads on the pins is also an important factor, and reviews have shown threads closer to the shoulder and half the length of the pin are highly recommended. A threaded pin increases heat generation and material velocity, leading to more mechanical interlocking between the materials to be joined [114]. Pin length also governs material flow and mixing. Reviews have suggested that the length of the pin should be such that the tip of the pin penetrates into the bottom sheet and the shoulder comes in contact with the top sheet without significant indentation that will lead to thinning of the top sheet. The P-FSSW tool produced stronger spot welds compared to a tool that had a pin penetrating the bottom sheet [115].

Based on the reviews carried out in Table 1, the following tool designs for FSSW are recommended to produce a sound weld joint. The tool design recommendations are based on the reviewed shapes and features, excluding factors such as ease of tool manufacturing, tool maintenance costs, and the effect of welding parameters on weld formation and quality.

**Figure 12:**
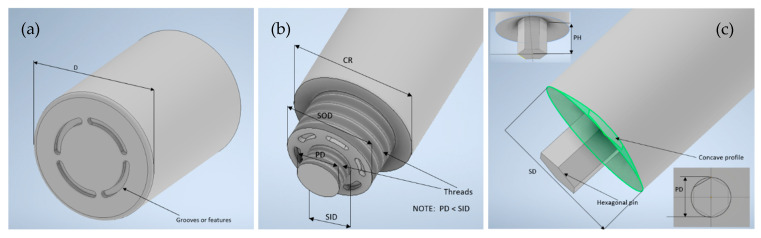
Recommended tool design for (**a**) P-FSSW, (**b**) R-FSSW, and (**c**) FSSW.

(i) P-FSSW: A concave-shaped tool (shoulder) with grooves or features such as involute or Fibonacci grooves and concentric features. P-FSSW creates fewer keyhole defects and produces spot welds with greater strengths compared to the use of a conventional FSSW tool [115]. P-FSSW tool is recommended for thin aluminum sheets (1 mm thick or less), as the conventional FSSW tool would create micro-defects on thin sheets [74]. The use of grooves in the P-FSSW tool assists with the penetration of the tool into the bottom sheet [116]. Referring to Table 2 and Figure 12a, the recommended and commonly used tool diameter is between 10 mm and 15 mm, and H13 or tungsten carbide is the tool material. D—pin-less tool shoulder diameter, M—tool material.

(ii) R-FSSW: Grooves or threads on the inner sleeve or the surface of the pin. The sleeve and pin are responsible for the plasticization of the materials due to heating and shearing and the circulation of the materials for weld formation [27]. The inclusion of grooves or threads in these components of the R-FSSW tool further enhance material mixing and deliver stronger weld development. However, the groove/thread in between the clamping ring and sleeve is prone to wear due to the relative motion between these two parts [26,27,119]. Referring to Table 3 and Figure 12b, the recommended and commonly used tool diameter is a clamping ring diameter between 15 mm and 18 mm, a sleeve diameter between 7 mm and 9 mm, and a pin diameter between 5 mm and 6 mm. H13 is the suggested tool material. CR—clamping ring diameter, SOD—sleeve outer diameter, SID—sleeve inner diameter, PD—pin diameter, M—tool material.

(iii) FSSW: Tool shoulder preferably with a concave profile and pin shapes such as hexagonal, triangular, tapered, and cylindrical, with threads and flutes. The threaded pin with flutes promoted significantly more material mixing and heat generation than the threaded pin [123], and the concave profile tool with the threaded pin improved material mixing and minimized particle segregation [124]. The pin length of the tool depends on the shoulder diameter and features added to the pin [115]. Referring to Table 4 and Figure 12c, the recommended and commonly used shoulder diameter ranges from 10 mm to 20 mm, while the pin diameter ranges from 3 mm to 8 mm. Steel is the suggested tool material for joining aluminum, copper, or polymers, and tungsten carbide is recommended for joining steels. SD—shoulder diameter, PD—pin diameter, PH—pin height, M—tool material.

## 7. Conclusions

This review studied the effect of FSSW tool geometry and design on spot weld formation. The following the conclusions are gained from this review:

- The heat generated during welding is a combined contribution from the frictional heat from the tool shoulder and the heat developed due to the plastic deformation and stirring effect by the tool pin.
- Bigger shoulder diameters and pin diameters increase the heat generation during welding.
- A concave shoulder profile produces a stronger weld compared to flat and convex profiles due to its ability to trap materials and transfer materials to the sheet interface efficiently for the development of a sound weld.
- Grooves such as Fibonacci and involute, and threads on P-FSSW and R-FSSW tools, also contribute to effective material flow during welding, hence assisting in heat generation and the development of a quality weld.
- The different shapes of pins, threads, and grooves on pins, and pin lengths, affect the materials’ plastic deformation and the flow of material upwards towards the tool shoulder.
- This review also provides recommendations on tool design for FSSW, P-FSSW, and R-FSSW.

## Figures and Tables

**Table 1:** FSSW tool components’ shapes and features.

Tool Component	Shapes	Features
Shoulder	(a) Flat (b) Concave (c) Convex	(a) Featureless (b) Concentric (c) Scrolled (d) Grooved
Pin/Probe	(a) Straight cylinder (b) Tapered cylinder (c) Square (d) Triangular (e) Hexagonal	(a) Threads (left-hand side and right-hand side) (b) Flutes (c) Flats (d) Stepped (e) Pin height

**Table 2:** Tool diameter and materials for P-FSSW.

Materials Joined	Tool Geometries and Material	References
DP590 and DP780 steels with 1.75 mm and 1.5 mm thickness, respectively.	D: 15.5 mm, flat shoulder. M: Tungsten–rhenium alloy	[19]
Aluminum alloys and copper with 0.5 mm thickness.	D: 10 mm, flat shoulder. M: H13	[67]
Aluminum alloys with 1 mm thickness.	D: 12 mm, flat and concave shoulders with grooves. M: H13	[68]
Aluminum alloys with 1.5 mm thickness.	D: 15 mm, flat featureless shoulders or with grooves. M: H13	[69]
Aluminum alloys with 1.8 mm thickness.	D: 15 mm, flat featureless shoulders or with grooves. M: not given	[70]
Copper with 1 mm thickness	D: 15 mm, flat shoulders with grooves. M: not given	[71]
Aluminum alloys with 2.0 mm thickness.	D: 10 mm, concave shoulder with grooves. M: H13	[72]
Magnesium alloys with 1.5 mm thickness.	D: 10 mm, with different profiles. M: Hardened steel	[73]
Aluminum alloys with 0.93 mm thickness.	D: 10 mm, featureless and with grooves. M: H13	[74]
CF/PA6 and aluminum alloy with 3 mm and 2 mm thickness, respectively.	D: 12 mm and 15 mm, flat and recessed. M: SKD61 steel	[75]
Aluminum alloys with 1.5 mm thickness.	D: 10 mm, with flat and 45° tapered edge. M: Tungsten carbide	[20]
Aluminum alloys with 4 mm thickness.	D: 24 mm, with flat shoulder. M: H13	[83]
Aluminum alloys with 3 mm thickness.	D: 12 mm, concave shoulder with grooves. M: H13	[116]
Aluminum and low-carbon steel with 0.93 mm and 1 mm thickness, respectively	D: 10 mm, flat featureless shoulders or with grooves. M: H13	[117]
Polyamide PA6 and aluminum alloy with 6 mm and 1 mm thickness, respectively.	D: 11 mm, flat shoulders with 0.5 mm fillet. M: Tungsten carbide	[118]

**Table 3:** Tool diameter and materials for R-FSSW.

Materials Joined	Tool Geometries and Material	References
Aluminum alloys with 1.8 mm thickness.	CR: 18 mm, SOD: 9 mm, SID: 6.4 mm, external thread and flat or 45° chamfer in the inner sleeve, PD: not given. M: not given	[76]
Aluminum alloys with 0.5 mm and 2.0 mm thickness.	CR: 14.5 mm, SOD: 9 mm, SID: not given, external threads and flat or trapezoidal grooves in the inner sleeve, PD: 6.4 mm. M: H13	[77]
Aluminum alloys with 2 mm thickness.	CR: 14.5 mm, SOD: 9 mm, SID: not given, external threads and flat or trapezoidal grooves in the inner sleeve, PD: 6.4 mm. M: H13	[120]
Aluminum alloys with 1.27 mm thickness.	CR: 18 mm, SOD: 9 mm, SID: not given, external threads and grooves in the inner sleeve, PD: 5.2 mm. M: H13	[78]
Aluminum alloys with 2 mm thickness.	CR: 18 mm, SOD: 9 mm, SID: not given, external threads and grooves in the inner sleeve, PD: 5.3 mm. M: SKD61 steel	[80]
Aluminum alloys with 1.2 mm and 2 mm thickness.	CR: not given, SOD: 9 mm, SID: 5.2 mm, with and without external threads and grooves or profiles in the inner sleeve, PD: 5.0 mm. M: not given	[81]
Aluminum and mild carbon steel with 1 mm and 1.2 mm thickness, respectively	CR: not given, SOD: 8 mm, SID: not given, three different taper position on inner sleeve, PD: 4.5 mm. M: Tungsten carbide	[82]
Aluminum alloys with 0.8 mm and 1.6 mm thickness.	CR: 17 mm, SOD: 9 mm, SID: 5.3 mm, with external grooves on sleeves, PD: 5.2 mm. M: Not given	[121]
Aluminum alloys with 1.6 mm thickness.	CR: 14.5 mm, SOD: 9 mm, SID: not given, with external grooves on sleeves, PD: 6 mm. M: Molybdenum vanadium tool steel	[27]
Aluminum alloys with 1.6 mm thickness.	CR: 15 mm, SOD: 7 mm, SID: 4.45 mm, PD: 4.40 mm. M: H13	[122]
Aluminum alloys with 2 mm thickness.	CR: 18 mm, SOD: 9 mm, SID: not given, threaded sleeve, PD: 5.2 mm and threaded. M: H13	[18]
Aluminum alloys with 1.5 mm and 1.6 mm thickness.	CR: 18 mm, SOD: 9 mm, SID: not given, threaded sleeve, PD: 6.4 mm and threaded. M: hot work tool steel	[26]
Aluminum alloys with 1.5 mm and 2.0 mm thickness.	CR: 18/15 mm, SOD: 7/5 mm, SID: not given, threaded sleeve, PD: 4/2.5 mm and threaded. M: hot work tool steel	[42]

**Table 4:** Tool diameter and materials for FSSW.

Materials Joined	Tool Geometries and Material	References
DP590 and DP780 with 1.75 mm and 1.5 mm thickness, respectively.	SD: 15.5 mm, PD: 4 mm, PH: 1.75 m/1.5 mm, concave shoulder, and conical pin. M: Tungsten–rhenium alloy	[19]
Polyethylene (HDPE) with 4 mm thickness.	SD: not given, PD: 7.5–10 mm, PH: 4–9 mm, flat and concave shoulders, and cylindrical and conical pins. M: Mild steel	[34]
Polyethylene (HDPE) with 4 mm thickness.	SD: 15–35 mm, PD: 5–11.25 mm, PH: 4–7 mm, concave shoulder, and cylindrical (threaded), tapered, square, triangular, hexagonal pins. M: 1040 steel	[63]
Aluminium alloy and copper with 0.5 mm thickness.	SD: 10 mm, PD: 3.3 mm/4.95 mm, PH: 0.2 mm/0.4 mm, flat shoulder, and cylindrical pin. M: H13	[67]
Aluminium alloy with 2.0 mm thickness.	SD: 10 mm, PD: not given, PH: 2 mm and 3 mm, concave shoulder, and cylindrical pins with M4 threads. M: H13	[72]
Aluminum alloys with 4 mm thickness.	SD: 24 mm, PD: 8 mm and 4 mm, with concave shoulder and conical pins. M: H13	[83]
Acrylonitrile butadiene styrene (ABS) with 6 mm thickness.	SD: 24 mm, PD: 9.2 mm, PH: 8 mm, flat shoulder, and flat, cylindrical, triangular, and tri-flute pins. M: 1045 steel	[84]
Aluminum alloys with 1.8 mm thickness.	SD: 12/16 mm, PD: 3/4 mm, PH: 2.6 mm, flat shoulder, and cylindrical and conical pins. M: H13	[86]
Aluminum alloys with 3 mm thickness.	SD: 12 mm, PD: 5 mm and 4/6/8 mm, PH: 5 mm, flat shoulder, and cylindrical and stepped pins. M: H13	[88]
Polycarbonate (PC) with 4 mm thickness	SD: 12 mm, PD: 5 mm and 4/6/8 mm, PH: 5 mm, flat shoulder, and cylindrical and stepped pins. M: Stainless steel	[90]
Aluminum alloys and thickness not given.	SD: 12 mm, PD: not given, PH: 1.7 mm, flat shoulder, and triangular, square, and circular pins. M: Not given	[91]
Aluminum alloys with 0.5 mm thickness.	SD: 10 mm, PD: 4.95 mm, PH: 0.2/0.4 mm, flat shoulder, and cylindrical, conical, triangular, and hexagonal pins with grooves and threads. M: H13	[92]
Aluminum alloys with 3 mm thickness.	SD: 15 mm, PD: 6 mm, PH: 3.5 mm, concave shoulder, and triangular, square, and circular pins. M: H13	[93]
Copper with 3 mm thickness.	SD: 18 mm, PD: 5 mm, PH: not given, flat shoulder, and cylindrical pin. M: H13	[125]
Aluminum and copper with 1.6 mm thickness.	SD: 15 mm, PD: 3 mm, PH: not given, flat shoulder, and cylindrical pin. M: H13	[126]
Aluminum and copper with 1.5 mm and 0.5 mm thickness, respectively.	SD: 6 mm, PD: 1.5 mm, PH: 0.6 mm, flat shoulder, and cylindrical pin. M: High-speed steel	[127]

## Data Availability

Add the data were included in the manuscript.
